# Tetraspanin CD9 affects HPV16 infection by modulating ADAM17 activity and the ERK signalling pathway

**DOI:** 10.1007/s00430-020-00671-5

**Published:** 2020-05-08

**Authors:** Snježana Mikuličić, Anna Fritzen, Konstanze Scheffer, Johannes Strunk, Carlos Cabañas, Maria Sperrhacke, Karina Reiss, Luise Florin

**Affiliations:** 1 Institute for Virology and Research Center for Immunotherapy (FZI), University Medical Center of the Johannes Gutenberg-University Mainz, Obere Zahlbacher Strasse 67, Augustusplatz, 55131 Mainz, Germany; 2 Department of Cell Biology and Immunology, Centro de Biología Molecular Severo Ochoa (CSIC-UAM), 28049 Madrid, Spain; 3 Department of Immunology, Ophthalmology and Otorhinolaryngology (IOO), Faculty of Medicine, Universidad Complutense, 28040 Madrid, Spain; 4 Instituto de Investigación Sanitaria Hospital 12 de Octubre (i+12), 28041 Madrid, Spain; 5 Department of Dermatology and Allergology, University Hospital Schleswig-Holstein Campus, Rosalind-Franklin-Straße 9, 24105 Kiel, Germany; 6 Max Planck Graduate Center, Mainz, Germany

**Keywords:** Papillomavirus, HPV, Infection, Entry, Receptor, L1, CD9, ADAM17, Tetraspanin, TSPAN29

## Abstract

Human papillomaviruses (HPV) are causative agents of various tumours such as cervical cancer. HPV binding to the cell surface of keratinocytes leads to virus endocytosis at tetraspanin enriched microdomains. Complex interactions of the capsid proteins with host proteins as well as ADAM17-dependent ERK1/2 signal transduction enable the entry platform assembly of the oncogenic HPV type 16. Here, we studied the importance of tetraspanin CD9, also known as TSPAN29, in HPV16 infection of different epithelial cells. We found that both overexpression and loss of the tetraspanin decreased infection rates in cells with low endogenous CD9 levels, while reduction of CD9 expression in keratinocytes that exhibit high-CD9 protein amounts, led to an increase of infection. Therefore, we concluded that low-CD9 supports infection. Moreover, we found that changes in CD9 amounts affect the shedding of the ADAM17 substrate transforming growth factor alpha (TGFα) and the downstream phosphorylation of ERK. These effects correlate with those on infection rates suggesting that a specific CD9 optimum promotes ADAM17 activity, ERK signalling and virus infection. Together, our findings implicate that CD9 regulates HPV16 infection through the modulation of ADAM17 sheddase activity.

**Electronic supplementary material:**

The online version of this article (10.1007/s00430-020-00671-5) contains supplementary material, which is available to authorized users.

## Introduction

Human papillomaviruses (HPVs) are small non-enveloped DNA viruses. Apart from causing a latent infection of basal keratinocytes, HPV is also known for inducing benign warts and diverse cancers such as anal, cervical, vaginal, vulvar, penile, and rectal cancer, as well as head and neck tumours [1, 2]. HPV high-risk types, including the most oncogenic type HPV16, are causative agents of cervical cancer. This kind of tumour is known as the most prevalent viral infection of the female genital tract with ranking as the fourth most frequent cancer in women [3, 4]. HPVs are composed of an icosahedral capsid harbouring the major capsid protein L1 and the minor capsid protein L2, and a circular double-stranded DNA genome [5].

For initial infection, virus particles require micro-lesions in the skin or mucosa to access lower, basal layers with residing stem cells [1, 2]. Here, heparan sulphate proteoglycans and laminins are identified as attachment receptors [6, 7]. Subsequent virus entry into the infectious pathway requires signalling processes [8–12] and virus association with secondary entry receptor molecules including tetraspanin CD151 [12–14], integrin complexes [14, 15], growth factor receptors (GFRs) [11, 12], and the phospholipid-binding protein annexin A2 [16, 17]. We have previously uncovered that not only CD151 but also additional tetraspanin family members, tetraspanin CD63 [18] and CD9 [19], act as proviral host-cell factors in HPV16 infection and that the cellular protease “A Disintegrin And Metalloprotease 17” (ADAM17), triggers the assembly of the HPV16 entry platform by modulating the extracellular signal-regulated kinases (ERK1/2) signalling pathway [12].

Tetraspanins are highly conserved and widely expressed transmembrane proteins that control numerous cellular processes including cell migration, adhesion, signal transduction, and protein trafficking [20, 21]. These molecules are announced as plasma membrane “master organizers” due to their ability to interact with multiple plasma membrane molecules and to form functional tetraspanin-enriched microdomains [21–23]. From the 33 tetraspanins identified in humans so far, only a portion has been shown to play an important role in regulating the entry process of various viruses including CD9, CD63, CD81 and CD151 [24, 25].

Like other members of the tetraspanin protein family, CD9 participates in the organization of tetraspanin microdomains through its lateral association with transmembrane proteins such as proteases, integrins and other adhesion proteins thereby modifying their localization and activity [26–30]. A direct association of CD9 with the metalloproteinase ADAM17 was reported in monocytic and endothelial cells. Through that interaction, CD9 was shown to negatively regulate ADAM17 shedding activity, resulting in reduced release of its substrates, the tumour necrosis factor *α* (TNFα) and the intercellular adhesion molecule 1 (ICAM-1) [31]. This functional interaction between CD9 and ADAM17 has been subsequently confirmed in other cell types and for additional ADAM17 substrates. In this regard, Tsukamoto et al. reported that CD9 negatively regulates the shedding of the substrate LR11, a member of the low-density lipoprotein receptor family which has a key role in cell migration, adhesion, and drug resistance, in various leukaemia cell lines [32]. Furthermore, Liu et al. have recently shown the direct association of CD9 with ADAM17 in keratinocytes and confirmed that CD9 exerts negative regulatory effects on this metalloproteinase resulting in diminished shedding of its substrate heparin-binding epidermal growth factor (HB-EGF) and reduced activation of EGFR/ERK signalling pathway, crucially affecting keratinocyte migration and wound healing [33].

In the context of host–pathogen interaction, CD9-enriched microdomains have been described as important host cell factors in infections by various viruses [24]. Likewise, our comparative analyses on the function of different tetraspanins and tetraspanin domains implicated a crucial role of CD9 in HPV16 infection of HeLa cells [19]. In this study, we investigate the functional relevance of tetraspanin CD9 in HPV16 infection of epithelial cells with different CD9 levels and the mechanistic details on how CD9 modulates virus entry.

## Materials and methods

### Cells

The human cervical carcinoma cell line (HeLa) was purchased from the German Resource Centre of Biological Material [(DSMZ), Braunschweig, Germany]. Human immortalized keratinocytes (HaCaT) were obtained from Cell Lines Services [(CLS), Eppelheim, Germany]. The cells were grown at 37 °C in Dulbecco’s modified Eagle’s medium [(DMEM), Invitrogen, Carlsbad, CA], supplemented with 1% Glutamax (Invitrogen), 10% foetal bovine serum [(FCS, Biochrom AG, Berlin, Germany)], 1% Eagle’s minimum essential medium (MEM) nonessential amino acids (GE Healthcare, Chicago, IL) and antibiotics (Fresenius Kabi, Bad Homburg vor der Hoehe, Germany). Cell lines were authenticated using Short Tandem Repeat (STR) analysis (Microsynth, Lindau, Germany) and tested negative for mycoplasma with Microsynth Real-Time PCR analysis (Microsynth, Lindau, Germany). Normal human epidermal keratinocytes (NHEK) were purchased from PromoCell (Heidelberg, Germany) and cultivated according to the manufacturer’s instructions.

### Production of pseudoviruses

HPV16 pseudoviruses (PsVs) were prepared as previously described [34–36]. Briefly, expression plasmids carrying codon-optimized HPV16 L1 and L2 cDNA (provided by Chris Buck; Bethesda, MD [34]) were cotransfected with a pcDNA3.1-Luciferase reporter plasmid into HEK 293TT (human embryonic kidney) cell line. Two days post-transfection, cells were lysed and PsVs were purified from the cell lysates by Optiprep (Sigma-Aldrich, St. Louis, MO) gradient centrifugation. Quantification of pcDNA3.1-Luciferase positive PsVs was performed as described [35, 36].

### Plasmids and antibodies

Human CD9 was amplified from pExpress-1-CD9, CD9 (clone IMAGp998A1815788Q, imaGenes, Berlin, Germany) by PCR and subcloned into the XhoI-KpnI site of the pEYFP-C1 (Clontech Laboratories, Mountain View, CA, USA) vector as described before [37] and into the XhoI-KpnI site of the pCMV-HA (Clontech) and pcDNA3.1/Hygro(−) (Thermo Fisher Scientific) vectors. The ADAM17 wild type (WT) plasmid was kindly provided by Dr. Gillian Murphy (Cambridge, UK) and was described previously [38]. Alkaline phosphatase (AP) tagged transforming growth factor α (TGFα-AP) was provided by Dr. Carl P. Blobel (Hospital for Special Surgery, New York, USA) [39].

The HPV16 L1-specific antibodies, mouse monoclonal antibodies (mAb) 33L1-7, 312F, and rabbit polyclonal antibody (pAb) K75, have been described previously [40–42]. The mouse mAb anti-CD9 (clone MM2/57) was obtained from Acris (Rockville, MD, USA), the mouse mAb anti-CD63 (sc-5275) from Santa Cruz (Dallas, TX, USA), the mouse mAb anti-HA (clone 16B12) from BioLegend (previously Covance, San Diego, CA, USA), and the pAb anti ADAM17 (Cat. #AB19027) from Merck Millipore (Darmstadt, Germany). β-actin (clone AC-15)- and α-tubulin (clone B-5–1-2)-specific mouse mAbs were obtained from Sigma-Aldrich. Rabbit monoclonal antibodies specific for total ERK1/2 (p44/42 MAPK; clone 137F5) and phosphorylated ERK (Phospho-p44/42 MAPK; clone D13.14.4E) were obtained from Cell Signaling (Leiden, Netherlands). Horseradish peroxidase-coupled (HRP) secondary antibodies for immunoblot were purchased from Jackson ImmunoResearch Europe Ltd. (Cambridgeshire, UK). Secondary antibodies (Alexa Fluor-conjugated) for immunofluorescence detection were obtained from Molecular Probes (Invitrogen).

### siRNA-mediated knockdown

The following *CD9*-specific siRNAs were obtained from Invitrogen: CD9#1 (CAAAGAGGUCUUCGACAAUAA) and CD9#2 (ACAAAUGUCUAUCAACUUUAA). AllStars Negative Control siRNA was used as nonsilencing control and was obtained from Qiagen (Hilden, Germany). Cells were transfected with 30 nM siRNA for 48 h using Lipofectamine RNAiMAX (Invitrogen) according to the manufacturer’s instructions.

### Overexpression

Cells were transfected with the indicated expression plasmids using Lipofectamine 2000 (Invitrogen) for 24 h. Afterwards, the cells were either processed for Western blot or exposed to HPV16 PsVs for immunofluorescence analyses and infection assays. For ectodomain shedding assay cells were transfected with polyethylenimine [(PEI), Sigma-Aldrich] for 24 h.

### Infection assays

The cells were exposed to ≈ 100 (HeLa and HaCaT) or ≈ 500 (NHEK) viral genome equivalents (vge) of PsVs per cell. One day after infection the cells were lysed with cell culture lysis reagent (Promega, Fitchburg, MA) and relative luciferase activity as gene transduction efficiency was measured using Luciferase substrate buffer (1 mM coenzyme A, 50 mM luciferin, 50 mM ATP, 0.5 M EDTA, 1 M DTT, 0.5 M Tris–HCl, pH 7.8, 1 M MgSO_4_) and normalized to lactate dehydrogenase (LDH) measurements (CytoTox-ONE Homogeneous Membrane Integrity Assay, Promega). Both luciferase and LDH activities were measured using the Tristar LB 941 luminometer (Berthold Technologies, Bad Wildbad, Germany).

### Ectodomain shedding assay

HeLa cells were transfected with a control plasmid or HA-CD9 plasmid in combination with AP-tagged ADAM17-substrate TGFα (TGFα-AP) using PEI or Turbofect as transfection reagent. On the day after, the cells were incubated with 200 ng/ml phorbol-12-myristate-13-acetate (PMA) for 2 h to stimulate ADAM17 [43]. Afterwards, supernatants were collected and the cells were lysed in lysis buffer containing 10 mM 1,10-phenanthroline, 1 mM EDTA and 2.5% Triton X-100 in water. Phenanthroline acts as a metalloprotease inhibitor of ADAM17 catalysis [44]. The AP activity was assessed after administration of AP substrate 4-nitrophenyl phosphate (Sigma-Aldrich) by measuring absorbance at 405 nm. The readout was performed on Multiskan RC V1.5–0 (Labsystems, Helsinki, Finland) and using GENESIS software. The AP activity in the supernatant was calculated in relation to total (supernatant and cell pellet) AP. HaCaT cells were transfected with CD9-specific siRNA and 24 h later with the TGFα-AP plasmid. The next day, the supernatants were collected and processed as described above. As HaCaT cells have high intrinsic ADAM17 activity, they did not require PMA-mediated ADAM17 activation.

### Cell binding assay

HeLas were transfected with *CD9*-targeting siRNAs, two days later detached, resuspended in serum-free DMEM, and transferred to siliconized reaction tubes. Next, they were incubated with HPV16 PsVs for 1 h, washed with phosphate-buffered saline (PBS) to remove unbound viruses, and then collected in sodium dodecyl sulfate (SDS) sample buffer (250 mM Tris–HCl, 0.3% glycerine, 0.1% SDS and 10% 2-mercaptoethanol) for Western blot.

### Western blot

For detection of the major capsid viral protein L1 and the HA tag, the cells were washed with PBS, lysed in SDS sample buffer supplemented with 2-mercaptoethanol, and denatured at 95 °C for 5 min. For CD9 detection with CD9-specific antibody, the cells were lysed in SDS sample buffer without 2-mercaptoethanol to preserve the epitope conformation and denatured at 95 °C. Afterwards, the samples were electrotransferred onto a nitrocellulose membrane (GE Healthcare) and blocked with 5% milk powder in PBS. The membrane was incubated with primary antibody at 4 °C overnight, the next day washed in PBST (phosphate-buffered saline containing 0.1% Tween-20), and stained with horseradish peroxidase (HRP)-conjugated secondary antibody. For detection of ERK proteins, the cells were lysed in lysis buffer containing 5 mM Tris–HCl pH 7.4, 1 mM EGTA, 250 mM sucrose, and 1% Triton X-100. The lysis buffer was supplemented with phosphatase inhibitor cocktail PhosSTOP (Roche) to prevent the degradation of phosphorylated proteins. Next, the cells were lysed applying three freeze–thaw cycles (freezing at − 80 °C and thawing at 4 °C) and denatured in SDS sample buffer at 95° for 5 min. The samples were electrotransferred onto nitrocellulose membrane and blocked with 5% milk powder in tris-buffered saline (TBS). After incubation with primary antibodies, the membrane was washed in TBST (tris-buffered saline containing 0.1% Tween-20) and proteins were detected with HRP-conjugated secondary antibody.

Detection was carried out using the Western Lightning Plus ECL detection reagent (PerkinElmer, Waltham, MA) and the signals were recorded on scientific imaging Super RX-N films (Fujifilm, Tokyo, Japan).

### Immunofluorescence

HeLa cells were transfected with siRNAs targeting CD9. Two days later, the cells were infected with HPV16 PsVs (with ≈ 100 particles per cell) and incubated at 37 °C for 7 h. Subsequently, the cells were fixed with 100% methanol and processed for staining with mAb L1-7 as described previously [13]. This mAb recognizes a specific epitope located in the interior of the pseudovirion capsid and is not accessible in intact virions. The samples were analysed by fluorescence microscopy using a Zeiss Axiovert 200 M inverted microscope equipped with a Plan-Apochromat 100x (1.4 NA) (Carl Zeiss, Jena, Germany) and quantified by ImageJ software (https://imagej.nih.gov/ij/). For quantification, the internalized particles were determined based on the L1-7 positive pixels relative to the cell nucleus signal (DNA/Hoechst 33342-positive pixels). Quantification was performed by analysis of at least 20 images (3–5 cells per image).

### Statistics

Statistical analyses were performed with GraphPad Prism 8.2.1 for Windows (GraphPad Software, San Diego, California USA, www.graphpad.com). Details of performed statistical assays are stated in the figure legends. Differences between the groups were considered statistically significant when *p* ≤ 0.05 with the statistical significance marked in the graph (*p ≤ 0.05, **p ≤ 0.01, ***p ≤ 0.001, *ns* not significant). All experiments were repeated independently at least three times if not stated otherwise.

## Results

### Primary binding of HPV16 PsV to the cell surface is independent of CD9

**Fig. 1:**
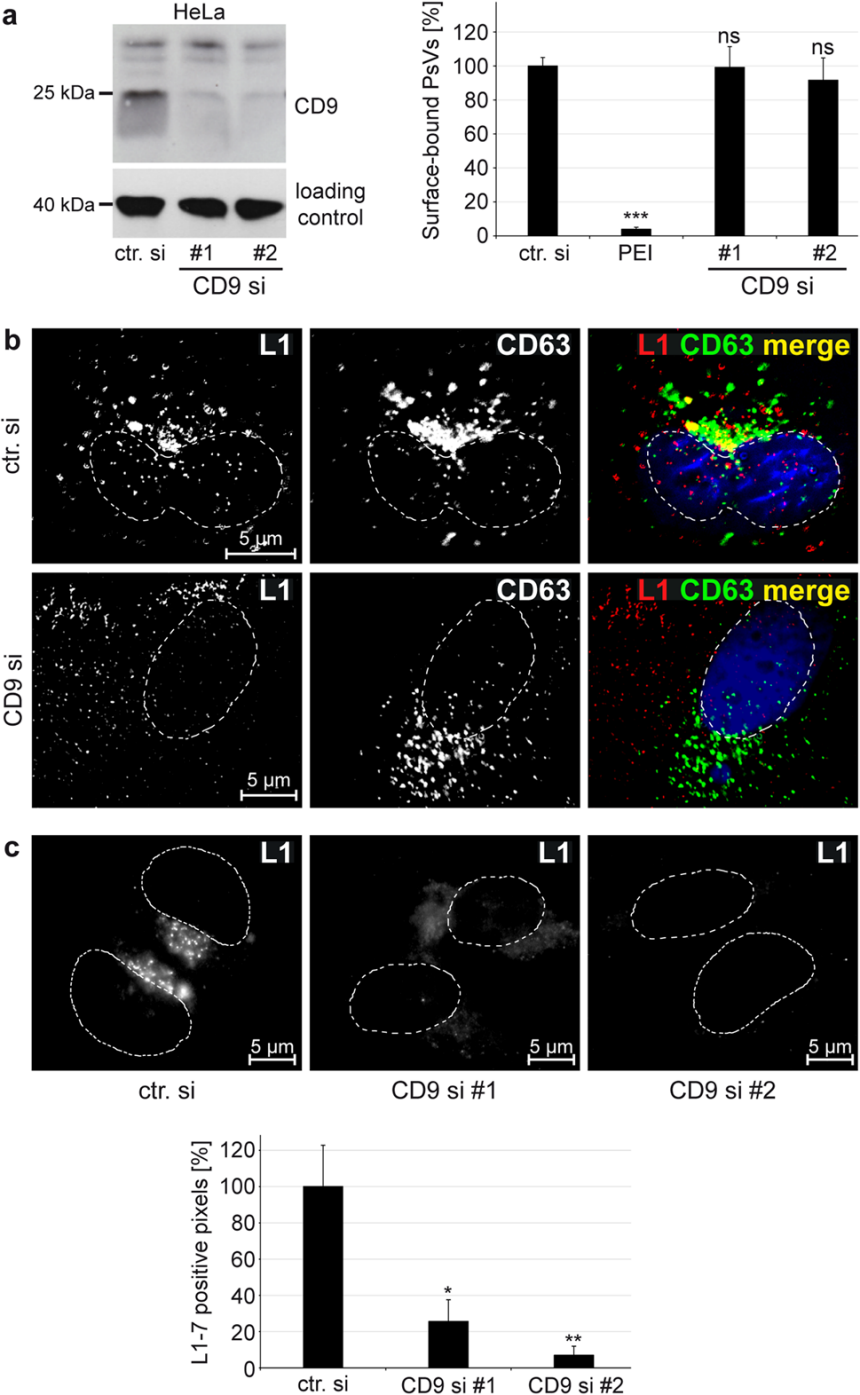
CD9 acts during the early trafficking of HPV16. **a** Primary binding of HPV16 PsV to the cell surface is independent of CD9. HeLa cells were transfected with control or CD9-specific siRNAs for 48 h. The efficiency of CD9 depletion is shown by Western blot under nonreducing conditions using CD9-specific mouse mAb. β-actin served as a loading control for protein input (left panel). After two days of siRNA treatment, cells were incubated with HPV16 PsVs for 1 h. Polyethylenimine (PEI) served as a positive control as it inhibits virus binding to the cell surface. Cell-bound PsVs were detected in cell lysates by Western blot using L1-specific mAb 312-F. The mean for control siRNA-treated cells (ctr. si) was set to 100% ± SEM. The data were compared to ctr. si and analysed with one-way ANOVA statistical assay (right panel) (*p ≤ 0.05, **p ≤ 0.01, ***p ≤ 0.001, ns = not significant). **b** CD9 depletion interferes with HPV16 trafficking to CD63 positive endosomes. Representative deconvoluted images of HeLa cells treated either with control or CD9-specific siRNA. 48 h after siRNA transfection the cells were exposed to HPV16 PsVs for 7 h, fixed and stained with anti-L1 pAb K75 (L1) and anti-CD63 (CD63) specific antibodies. Nuclei were stained with Hoechst 33,342 and are shown in blue or with a dashed line. **c** CD9 depletion restricts capsid disassembly. Representative images (upper panel). HeLa cells were treated either with control or CD9-specific siRNAs for 48 h and infected with HPV16 PsVs for 7 h. Virus uptake into the cell was analysed by immunofluorescence using mAb L1-7. This antibody recognizes an epitope accessible only after virus capsid disassembly. Nuclei are depicted with dashed lines. Quantification of immunofluorescence images (lower panel). L1-7 positive pixels were analysed using ImageJ. The mean for control siRNA-treated cells (ctr. si) was set to 100% ± SEM. The data were compared to ctr. si and analysed with ordinary one-way ANOVA statistical assay (*p ≤ 0.05, **p ≤ 0.01, ***p ≤ 0.001, *ns* not significant).

Current data suggest that the tetraspanin CD9 plays an important role in HPV16 pseudovirus (PsVs) infection of HeLa cells [19]. To further determine the role of CD9 during the HPV16 entry pathway, we first analysed the influence of CD9 depletion on virus-cell binding efficiency, virus trafficking and capsid disassembly in HeLa cells. While polyethylenimine (PEI), a potent inhibitor of virus-cell interaction [35], almost entirely blocked the binding of HPV PsVs to the cell surface no influence of CD9 siRNA treatment was observed (Fig. 1a).

### Post-binding steps of HPV16 entry require CD9

To follow HPV16 during the early steps of virus entry, we performed immunofluorescence stainings of the major capsid protein L1 and CD63, a tetraspanin that accumulates with HPV16 capsids in multivesicular bodies (MVBs) [18, 45]. As earlier described, control siRNA-treated HeLa cells showed prominent HPV16-CD63 colocalization in vesicular structures (Fig. 1b, upper panel). By contrast, CD9-depleted cells showed only a minor colocalization of L1 and CD63 while viral capsids localized in the cell periphery (Fig. 1b, lower panel) suggesting that HPV16 uptake and trafficking to MVBs are disturbed. To verify the crucial role of CD9 in regulating early HPV16 trafficking, a prerequisite for capsid disassembly, we examined the quantity of disassembled capsids in endosomes. Here, we used the disassembly-specific monoclonal antibody L1-7 that recognizes a linear L1-epitope, which is only accessible after capsid disassembly [13]. CD9 knockdown led to a strong reduction of L1-7 positive pixels (Fig. 1c) again demonstrating the functional relevance of CD9 for HPV16 entry.

Our findings indicate that CD9 acts early in the HPV16 entry pathway after the primary attachment of viral particles to the cell surface.

### Depletion of CD9 leads to cell-line specific effects on HPV16 pseudovirus infection rate in human epithelial cells

**Fig. 2:**
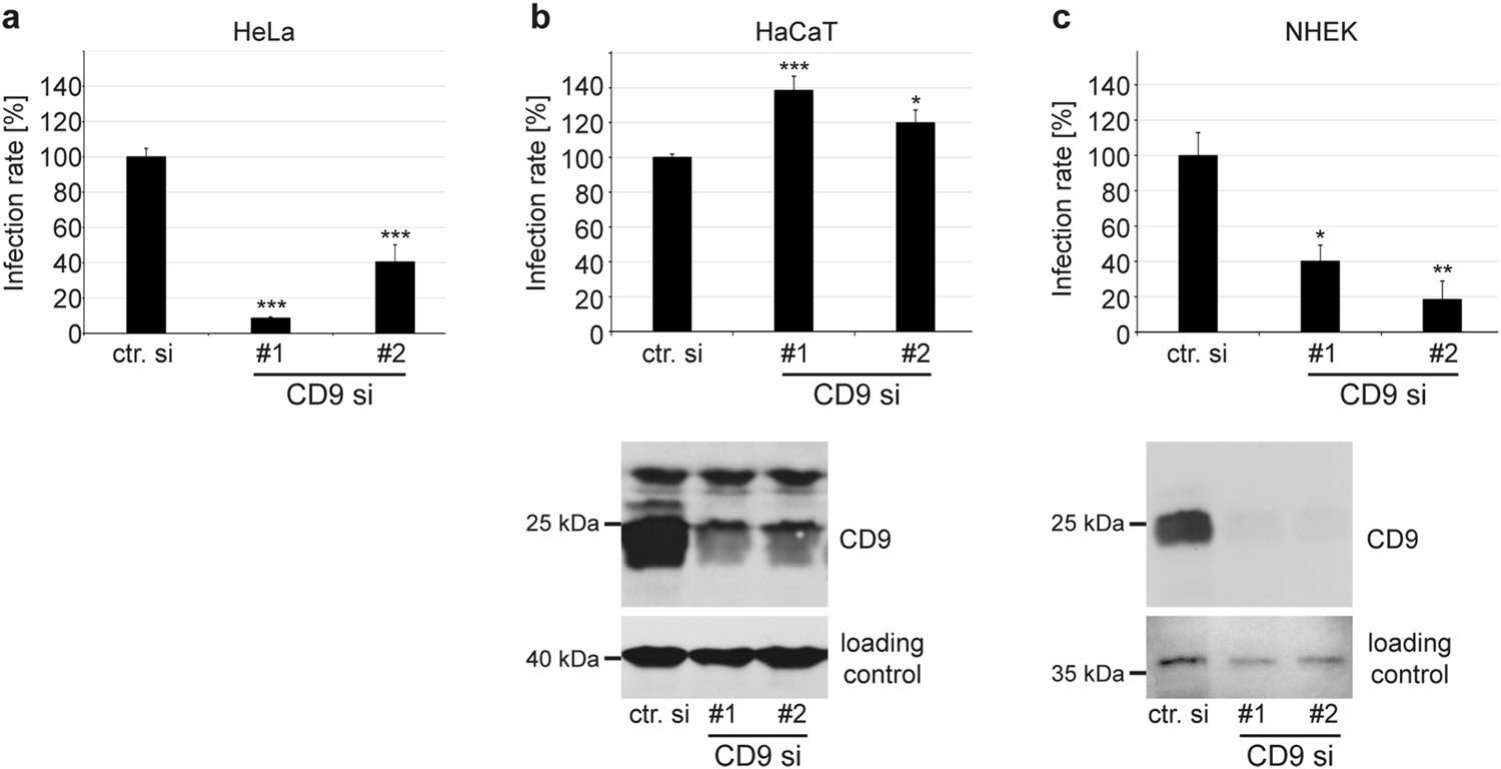
Depletion of CD9 leads to cell-line specific effects of HPV16 PsV infection rate in HeLa, HaCaT cell lines and primary keratinocytes (NHEK). HeLa (**a**), HaCaT (**b**), and NHEK (**c**) cells were transfected with two CD9-specific siRNAs (#1 and #2) for 48 h. Lower panels show the efficiency of CD9 depletion in HaCaT and NHEK. Western blot was performed as described and shown for HeLa in Fig. 1a. β-actin or nonspecific band produced by CD9-specific mouse mAb served as a loading control for protein input in blots with HaCaT and NHEK, respectively. Upper panels show assessed infection rates. Two days after siRNA transfection, the cells were treated with HPV16 PsVs and the infection rate, measured by luciferase activity, was assessed 24 h later. Luciferase counts were normalized by lactate dehydrogenase (LDH) counts. The mean of the infection rate after control siRNA (ctr. si) treatment was set to 100% ± SEM. Data were compared to the control and for **a** and **b** analysed with ordinary one-way ANOVA, while for **c** with Kruskal–Wallis statistical assay (*p ≤ 0.05, **p ≤ 0.01, ***p ≤ 0.001, *ns* not significant)

To substantiate the role of CD9 in HPV infection of human epithelial cells we tested the effect of CD9 protein knockdown on HPV16 PsVs infection rate of HeLa, HaCaT and primary keratinocytes (NHEK) (Fig. 2). Surprisingly and in contrast to our findings in HeLa and NHEK cells, the infection rate was clearly enhanced in HaCaT cells (Fig. 2, upper panels), while *CD9*-specific siRNAs caused a strong reduction of total CD9 amounts in all cell lysates tested (Fig. 1a for HeLa, Fig. 2 lower panels for HaCaT and NHEK). This finding demonstrates that CD9 depletion causes cell-specific effects on HPV16 infection rate in different epithelial cells.

### HeLa, HaCaT and NHEK express different levels of CD9

**Fig. 3:**
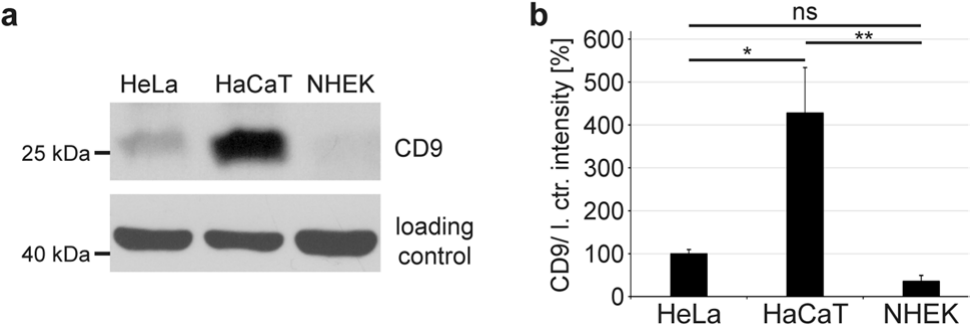
CD9 expression levels in HeLa, HaCaT and NHEK cells. **a** Western blot (WB) of CD9 expression in HeLa, HaCaT and NHEK cells. WB was performed under nonreducing conditions applying CD9 mouse mAb. **b** Quantification of WB bands displayed in **a**. The graph shows relative CD9 band intensities. β-actin served as a loading control for protein input. The mean of CD9 band intensities detected in HeLa cells was set to 100% ± SEM. The data were analysed with ordinary one-way ANOVA statistical assay (*p ≤ 0.05, **p ≤ 0.01, ***p ≤ 0.001, *ns* not significant)

To test whether endogenous CD9 expression levels influence infection rates in HeLa, HaCaT and NHEK cells, we initially compared endogenous CD9 protein amounts in cell lysates using quantitative analysis of Western blot bands (Fig. 3). Using equal protein amounts in the cell lysates and the same exposure times for all tested cell lines, we found that HaCaT cells exhibit a four- to ten-fold higher CD9 expression level, when compared to HeLa and NHEK cells, respectively. These results suggest that the contrary outcome in infection rates in HeLa and NHEK versus HaCaT cells is associated with the different intracellular expression level of CD9. Hereby, both a particularly high (HaCaT) and the absence of CD9 protein (CD9-siRNA depleted HeLa and NHEK) seemed to have a negative effect on the infection rate. Thus, we speculate that a low CD9 expression, as is the case for HeLa and NHEK cells in a natural state, promotes HPV infection.

### High CD9-expression represses HPV16 infection rate

**Fig. 4:**
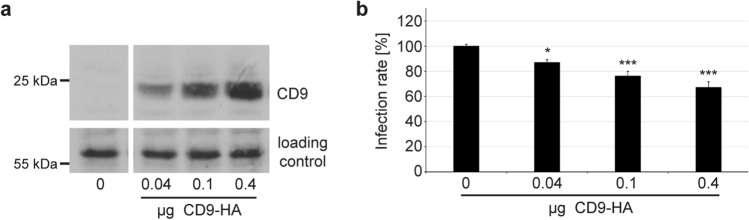
CD9 overexpression represses HPV16 PsV infection rate in a dose-dependent manner. HeLa cells were transfected with increasing amounts of HA-tagged CD9 expression plasmid, including 0 µg in the control, 0.04, 0.1 and 0.4 µg for 24 h. A control plasmid was cotransfected for a total amount of 0.4 µg transfected DNA. **a** To control CD9 expression levels WB was performed under nonreducing conditions. The nonspecific band produced by the anti-HA antibody served as a control for protein input. **b** One day after plasmid transfection the cells were infected with HPV16 PsVs. The infection rate was measured 24 h later by determining luciferase activity. The mean for control transfected cells (control; 0 µg HA-CD9) was set to 100% ± SEM. The data were compared to control transfected cells and analysed with ordinary one-way ANOVA statistical assay (*p ≤ 0.05, **p ≤ 0.01, ***p ≤ 0.001, *ns* not significant)

To further investigate this hypothesis, we transfected low-expressing HeLa cells with different amounts of expression plasmids encoding for HA-tagged CD9 (Fig. 4) or untagged CD9 (Fig. S1). These experiments confirmed that an increase of CD9 in significantly impairs HPV16 PsVs infection in a dose-dependent manner.

### A specific CD9 optimum promotes ADAM17 activity and ERK signalling

**Fig. 5:**
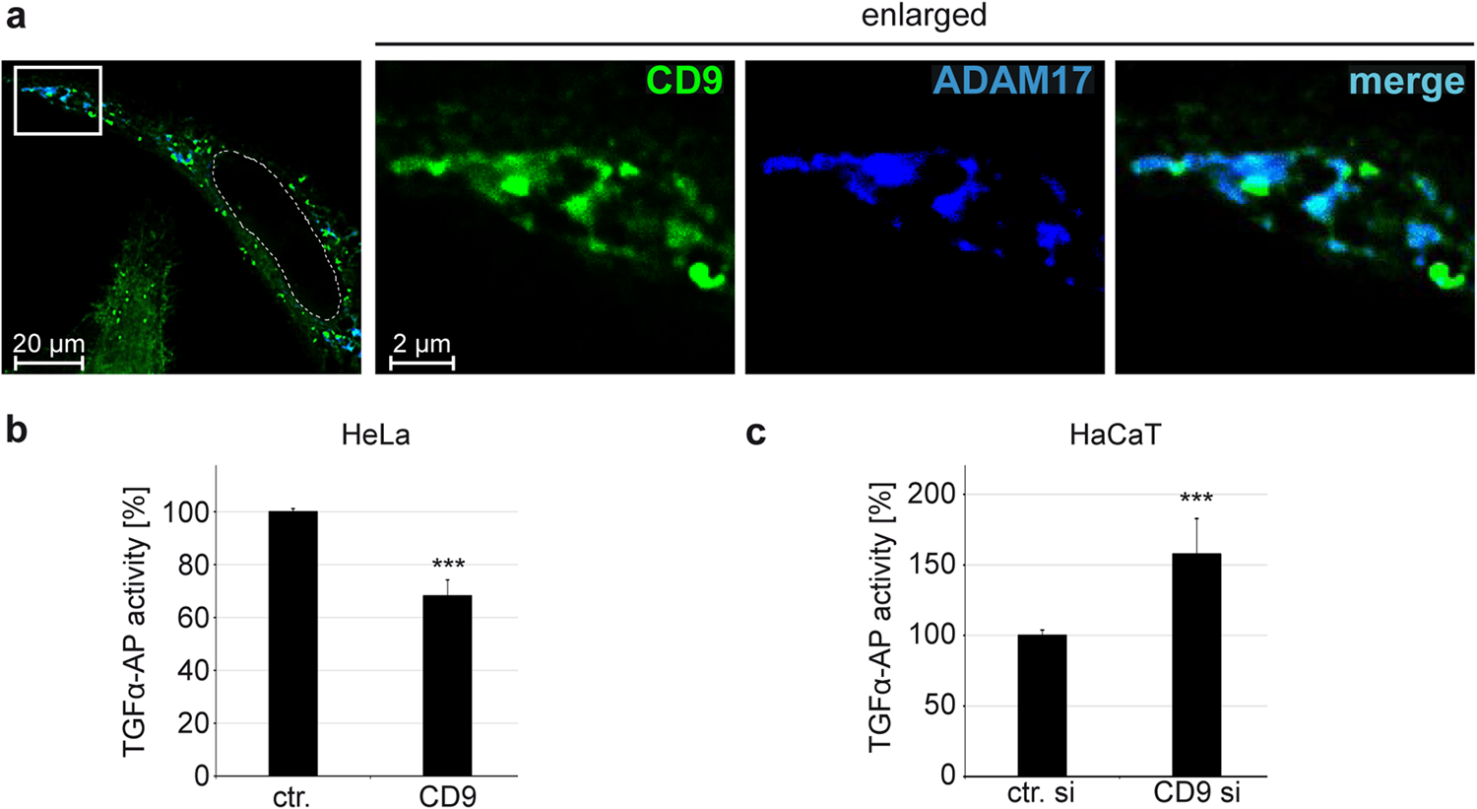
CD9 colocalizes with ADAM17 and inhibits the sheddase activity of the protease. **a** Representative images of HeLa cells cotransfected with YFP-tagged CD9 (displayed in green) and ADAM17 expression plasmids for 24 h and subsequently stained with ADAM17-specific antibody (blue). The nucleus was stained with Hoechst 33,342 and is depicted with a dashed line. **b** CD9 overexpression diminishes ADAM17-mediated TGFα release. HeLa cells were cotransfected with control or HA-tagged CD9 plasmid and alkaline phosphatase-tagged TGFα (TGFα-AP) plasmids for 24 h. The next day cells were stimulated with phorbol 12-myristate 13-acetate (PMA) for 2 h. Assessed AP activity served as a measure for ADAM17-mediated release of TGFα-AP. AP activity is shown as a percentage and the mean for control plasmid-transfected cells (ctr.) was set to 100% ± SEM. The data were analysed with two-tailed unpaired *t* test (*p ≤ 0.05, **p ≤ 0.01, ***p ≤ 0.001, *ns* not significant). **c** CD9 depletion increases ADAM17-mediated TGFα release. HaCaT cells were depleted of CD9 and the next day transfected with alkaline phosphatase-tagged TGF-α (TGF-α-AP) plasmid for 24 h. The next day AP activity was assessed and served as a measure for released ADAM17-specific substrate TGFα-AP. AP activity is shown as a percentage and the mean for control siRNA-transfected cells (ctr. si) was set to 100% ± SEM. The data were analysed with two-tailed unpaired *t* test (*p ≤ 0.05, **p ≤ 0.01, ***p ≤ 0.001, *ns* not significant)

Furthermore, it has been well established that CD9 also regulates the function of cell membrane proteases including ADAM17 [28, 30, 31, 33]. ADAM17, in turn, triggers ERK signalling which is important for HPV16 entry receptor formation [12]. Therefore, we speculated that CD9 affects HPV infection by modulating ADAM17 activity and thus analysed the spatial distribution and association of the two plasma membrane proteins in HeLa cells (Fig. 5a). In agreement with earlier studies [31, 33], our immunofluorescence analyses demonstrated microscopic overlap between CD9 and ADAM17.

**Fig. 6:**
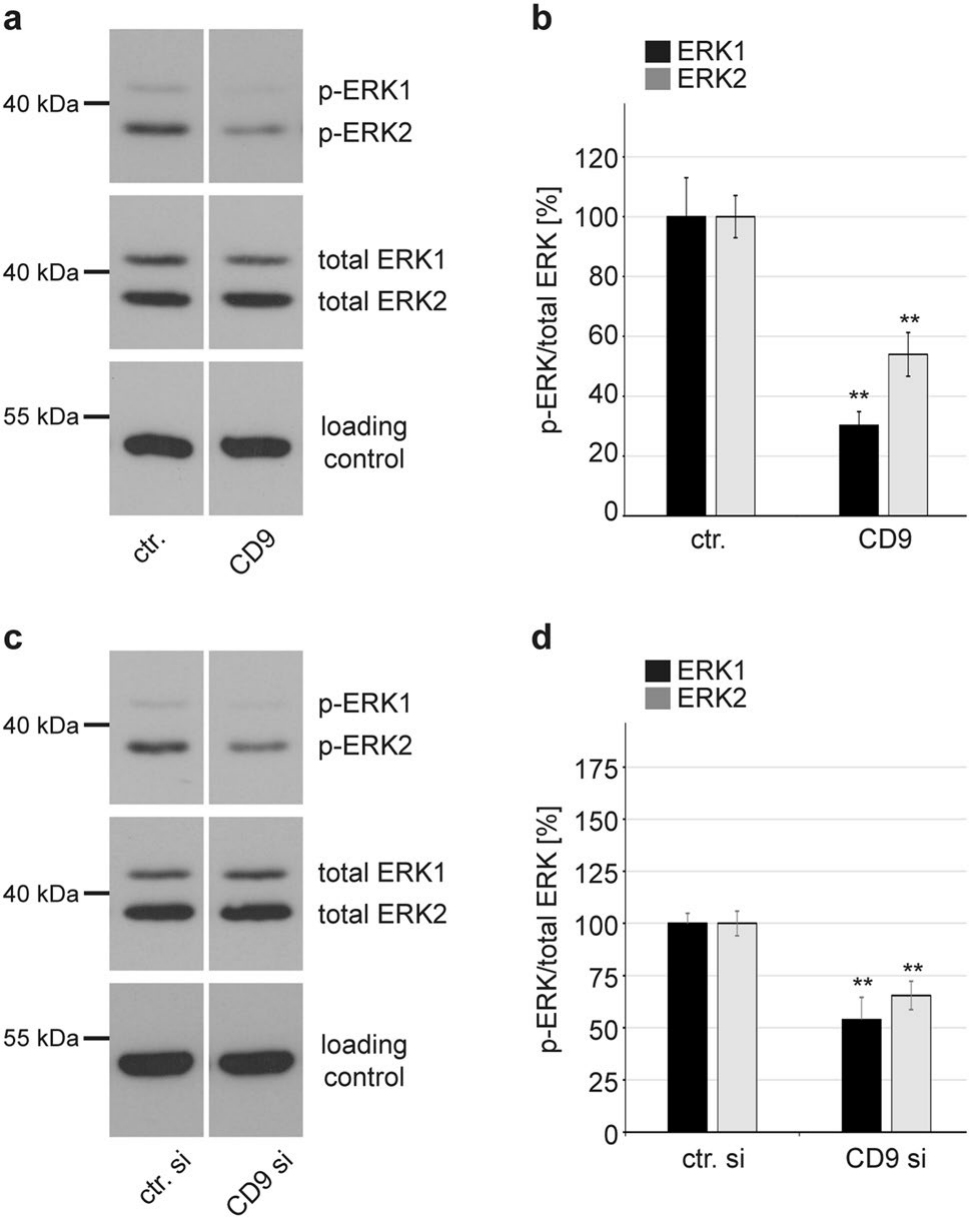
CD9 overexpression and depletion reduce ERK1/2 phosphorylation in HeLa cells. **a** HeLa cells were transfected either with control (ctr.) or with CD9-expression plasmid for 24 h. The next day cells were starved for 1 h in medium without FCS and afterwards, the lysates were collected. **b** Quantification of WB shown on **a**. **c** HeLa cells were transfected either with control (ctr. si) or with CD9-specific siRNA. Two days later cells were starved for 1 h in medium without FCS and the lysates were collected. **d** Quantification of WB shown on **c**. **a**–**d** WB shows phosphorylated and total ERK1 and ERK2 for the indicated conditions; α-tubulin was used as a loading control. The amount of phosphorylated ERK1 (black) and ERK2 (grey) is shown as a ratio of phosphorylated to total ERK form. The values are given as mean ± SEM and the mean for ERK1 and ERK2 for the control was set to 100%. Data for ERK1 and ERK2 after CD9 manipulation compared to the corresponding ERK1 and ERK2 of the control condition were analysed with two-tailed unpaired *t* test (*p ≤ 0.05, **p ≤ 0.01, ***p ≤ 0.001, *ns* not significant). Overexpression experiments were repeated independently at least two times with total *n* = 6 for control and *n* = 5 for CD9

Next, we analysed the regulatory effect of CD9 on ADAM17 activity in HeLa and HaCaT cells. A TGFα-AP shedding assay was used to report ADAM17 activity. This well-defined ADAM17 substrate [39, 46, 47], the membrane-bound transforming growth factor alpha (TGFα) was fused to the alkaline phosphate (AP) and quantification of the AP activity in the supernatant and cell lysates served as a measure for ADAM17 activity. Our data uncovered a significant decrease in the amount of released TGFα-AP into the supernatant of HeLa cells after CD9 overexpression (Fig. 5b). This effect was comparable to its effect on HPV16 PsV infection as shown above (Fig. 4). Conversely, depletion of CD9 in HaCaT cells led to a significant increase in the amount of released TGFα-AP (Fig. 5c), which again corresponds to the effect of CD9 siRNA on infection rates in these cells.

As CD9 influences ADAM17-activity [31–33, 48] as well as ERK signalling [49, 50] (see also Kummer et al., in the same issue [62]), and ADAM17 regulates HPV16 infection via the ERK1/2 signalling pathway [12], we expected that the cellular CD9 level likewise would influence ERK1/2 activation in our cell model. Indeed, modulation of the endogenous CD9 level in HeLa cells resembles the results obtained in HPV16 PsVs infection assays (for infection assays in CD9 overexpressing HeLas see Figs. 4b and S1; for knockdown see Fig. 2a). This was investigated by overexpressing and depleting CD9 in HeLa cells and we observed that both treatments induced a significant decrease of phosphorylated ERK1 and ERK2 levels (Fig. 6). Our results imply that a low CD9 level supports ADAM17-mediated ERK activation and consequently HPV16 entry and infection.

## Discussion

In the present study, we demonstrate the CD9-regulated modulation of HPV16 entry and infection of human epithelial cells. More specifically, a low CD9 expression level supports infection as well as ADAM17 sheddase activity and the ADAM17-mediated ERK signalling pathway. As both processes, the activity of the sheddase and the downstream ERK signalling, are required for the assembly of the HPV entry platform formation [12], the data suggest that the effects are associated; or more precisely, that CD9 affects HPV16 entry by modulating ADAM17 activity.

Initial experiments showed that CD9 is involved after virus primary attachment to the cell surface and before capsid disassembly occurs. Inhibited CD63 colocalization as well as blocked capsid disassembly were both observed after CD9 knockdown and are strong indications of disturbed virus entry as also observed after depletion of other crucial entry mediators such as the annexin A2/S100A10 heterotetramer (A2t) [51], the obscurin-like protein OBSL1 [52], or the tetraspanin CD151 [13, 14].

Surprisingly, depletion of CD9 decreased the infection rate in HeLa cell line and primary keratinocytes (NHEK), while in HaCaT cells the outcome was the opposite. Subsequent quantitative analysis on endogenous CD9 levels revealed that significantly lower amounts of CD9 are expressed in HeLa and NHEK when compared to HaCaT cells. This observation led to the speculation that a low level of endogenous CD9, as it is the case in nontreated HeLa and NHEK cells, promotes HPV16 infection, whereas high CD9 amounts, naturally present in HaCaT cells, impair HPV16 infection. This notion was further confirmed by the dose-dependent decrease of HPV16 infection in HeLa cells following CD9 overexpression, thereby mimicking the high CD9 levels naturally present in HaCaT cells.

As a member of the tetraspanin family, CD9 closely interacts with numerous membrane proteins. In particular, the direct interaction between CD9 and the transmembrane metalloproteinase ADAM17 has been reported in different cell types, including leukocytes, endothelial cells and keratinocytes [31–33, 48]. Through this direct association, CD9 has been shown to exert negative regulatory effects on the sheddase activity of ADAM17, which is responsible for ectodomain shedding from a large variety of substrate transmembrane proteins, including adhesion molecules, cytokines, chemokines, growth factor receptors, and multiple ERK-activating growth factors, including TGFα, HB-EGF, epiregulin and amphiregulin [30, 53–55].

We have confirmed here the association of CD9 and ADAM17 by their microscopic overlap in HeLa cells. Functional analyses revealed a repressive function of high CD9 expression levels on ADAM17 shedding activity in different epithelial cells. This is in line with the previously reported results showing CD9-mediated negative regulation of ADAM17 shedding activity in different cells [31, 33, 56]. Furthermore, modulation of the CD9 amounts, both through its increase or decrease, led to a reduction of ERK1/2 activation in HeLa cells. These effects correlate with those on infection rates. Moreover and similarly to our comparative infection analyses in different cells, opposing effects on ERK activation upon CD9 depletion were observed depending on the cell line under study [49, 50]. Differences in endogenous CD9 expression levels might explain these contradictory results and support our conclusions drawn from our infection data.

Since expression of CD9 has been shown not to affect the endogenous expression of ADAM17 in different cell types [33, 56], two alternative and non-mutually exclusive possibilities exist to explain our results: The first one is that CD9 regulates ADAM17 shedding activity through their direct association on the cell membrane. Second, that CD9-mediated compartmentalization of ADAM17 and its substrates might regulate substrate accessibility to the metalloproteinase.

Regarding the first possibility, it has been shown earlier that CD9-regulated impact on ADAM17 protease activity arises from a direct CD9-ADAM17 interaction that disables ADAM17-mediated cleavage, while CD9 dissociation enables ADAM17 activity [31]. The sheddase activity of ADAM17 has been proposed to be also regulated through other factors, such as interaction with integrin α5β1 [57, 58]. Associations between the sheddase and this integrin resulted in the inhibition of both the adhesive capacity of integrin α5β1 and the metalloproteinase activity of ADAM17 due to steric hindrance leading to the decreased accessibility of its catalytic site for the substrates. Moreover, the formation of tri-molecular complexes comprising CD9, ADAM17, and integrin α5β1 and interactions between laminin-binding integrins have been described [28, 56, 59]. Therefore, varying expression levels of CD9 could influence integrin localization and activity that might also contribute to the observed changes in HPV16 infection rates, as integrins are important proviral factors contributing to virus binding and signalling.

The second possibility is supported by findings showing that CD9 not only binds to ADAM17 but also to the ADAM17 substrates, the membrane-bound forms of GFs like heparin-binding EGF-like growth factor precursor (pro-HB-EGF), transforming growth factor alpha precursor (pro-TGFα) and pro-amphiregulin [26, 30, 31]. Likewise, during influenza A virus (IAV) and coronavirus (CoV) entry, CD9 mediates the close proximity of a protease and the virus enabling the cleavage of viral proteins which is a precondition for infection [24, 60, 61]. Similar concepts of the regulation of signaling pathways by tetraspanins is summarized in Kummer et al. in this issue [62]. Therefore, we propose that CD9 might regulate the local distribution of the protease ADAM17 and its EGFR-activating substrates, which then influences the organization of the HPV16 entry receptor platform and eventually infection.

In this model, CD9 microdomains would represent storage areas with increased density of ADAM17 and its substrates. As CD9 interacts directly with ADAM17 and with its EGFR-activating substrates, a high amount of CD9 in these microdomains would impose limitations in the movement of these molecules, restraining the accessibility of substrates to the catalytic site of ADAM17, thus resulting in their reduced shedding and EGFR/ERK activation required for HPV infection. Likewise, an almost complete loss of CD9 in the microdomain might enhance the free diffusion of both protease and substrates but at the cost of reducing their effective local concentrations on the plasma membrane required for the optimal accessibility of substrates to the catalytic site of ADAM17, again leading to impaired shedding. Only a low level of CD9 would then ensure the proper membrane composition for storage, diffusion, and accessibility of ADAM17 and its EGFR-activating substrates. Therefore, this low-CD9 expression level represents the CD9 optimum which enables efficient virus infection.

## Electronic supplementary material

Below is the link to the electronic supplementary material.

Supplementary file1 (DOCX 313 kb)
