# Supplementary material for: Tetraspanin CD9 affects HPV16 infection by modulating ADAM17 activity and the ERK signalling pathway
Source: Med Microbiol Immunol. 2020 May 8;209(4):461–71. doi: 10.1007/s00430-020-00671-5 (PMC7206579; doi:10.1007/s00430-020-00671-5)
Supplement: Supplementary file 1 — Supplementary file1 (DOCX 313 kb) [file 430_2020_671_MOESM1_ESM.docx]

**Supplementary figure**

**Fig. S1** CD9 overexpression represses HPV16 infection rate in a dose-dependent manner. HeLa cells were transfected with control plasmid or indicated concentrations of pcDNA3.1(-)/Hygro(+)-CD9 plasmid for 24 h (using 0.4 µg CD9 plasmid for expression control in the WB). (a) WB to control CD9 expression was performed under nonreducing conditions. The nonspecific band produced by the CD9 mouse mAb served as a control for protein input. (b) HeLa cells were either left nontransfected or transfected with CD9 plasmid amounts as indicated. One day later, the cells were infected with HPV16 PsVs and another 24 h later analysed. The infection rate was measured detecting luciferase activity that was normalized to LDH measurements. The mean for CD9-nontransfected cells (0) was set to 100% ± SEM. The data (compared to 0 µg of CD9 plasmid, denoted as 0) were analysed with Kruskal-Wallis statistical assay (p≤0.05 *, p≤0.01 **, p≤0.001 ***, ns = not significant).
